# Implementation of a medical student-run telemedicine program for medications for opioid use disorder during the COVID-19 pandemic

**DOI:** 10.1186/s12954-020-00438-4

**Published:** 2020-11-17

**Authors:** Marcus Castillo, Brianna Conte, Sam Hinkes, Megan Mathew, C. J. Na, Ainhoa Norindr, David P. Serota, David W. Forrest, Amar R. Deshpande, Tyler S. Bartholomew, Hansel E. Tookes

**Affiliations:** 1 Department of Medical Education, University of Miami Miller School of Medicine, Miami, FL USA; 2 Division of Infectious Diseases, Department of Medicine, University of Miami Miller School of Medicine, Miami, FL USA; 3 Department of Anthropology, College of Arts and Sciences, University of Miami, Miami, FL USA; 4 Department of Public Health Sciences, University of Miami Miller School of Medicine, Miami, FL USA

**Keywords:** COVID-19, Telehealth, Medications for opioid use disorder, Student-run clinic

## Abstract

**Objectives:**

The COVID-19 pandemic led to the closure of the IDEA syringe services program medical student-run free clinic in Miami, Florida. In an effort to continue to serve the community of people who inject drugs and practice compassionate and non-judgmental care, the students transitioned the clinic to a model of *TeleMOUD* (medications for opioid use disorder). We describe development and implementation of a medical student-run telemedicine clinic through an academic medical center-operated syringe services program.

**Methods:**

Students advertised *TeleMOUD* services at the syringe service program on social media and created an online sign-up form. They coordinated appointments and interviewed patients by phone or videoconference where they assessed patients for opioid use disorder. Supervising attending physicians also interviewed patients and prescribed buprenorphine when appropriate. Students assisted patients in obtaining medication from the pharmacy and provided support and guidance during home buprenorphine induction.

**Results:**

Over the first 9 weeks in operation, 31 appointments were requested, and 22 initial telehealth appointments were completed by a team of students and attending physicians. Fifteen appointments were for MOUD and 7 for other health issues. All patients seeking MOUD were prescribed buprenorphine and 12/15 successfully picked up medications from the pharmacy. The mean time between appointment request and prescription pick-up was 9.5 days.

**Conclusions:**

*TeleMOUD* is feasible and successful in providing people who inject drugs with low barrier access to life-saving MOUD during the COVID-19 pandemic. This model also provided medical students with experience treating addiction during a time when they were restricted from most clinical activities.

## Introduction

The coronavirus disease 2019 (COVID-19) global pandemic has highlighted inequalities that disproportionately affect marginalized communities, including persons who inject drugs (PWID) [1, 2]. PWID are more likely to have chronic medical conditions and experience homelessness, incarceration, and crowded living conditions compared to the general population, all which pose unique challenges regarding the transmission of the virus. Furthermore, increased stress and anxiety can worsen mental health comorbidities and lead to an increase in substance use [3].

The Infectious Disease Elimination Act (IDEA) syringe services program (SSP) at the University of Miami was established to improve the health outcomes of PWID by providing them with sterile injection equipment, naloxone, and improved access to medical care [4]. A free student-run clinic in collaboration with the Mitchell Wolfson Sr Department of Community Service (DOCS), operating out of the IDEA SSP, was developed to meet the medical needs of participants and create a space to educate students on the principles of harm reduction. The clinic’s primary function was to provide primary and specialty care to PWID, particularly general health screenings, referrals for medications for opioid use disorder (MOUD), and wound care for skin and soft tissue infections [5]. With the onset of COVID-19, 43% of SSPs nationally reduced their services and 25% reported closures [6]. Similarly, the IDEA SSP curtailed its hours of operation and services, and the on-site clinic was suspended.

In order to continue serving the local population of PWID and fostering educational patient interactions, students conceived and implemented a free virtual clinic that offers MOUD and addresses general health concerns. The students understood the benefits of this medication after having gone through the University of Miami Miller School of Medicine opioid education curriculum that was introduced in 2019 to train and educate all medical students on identifying and treating patients with substance use disorder. Funded by the Florida Department of Children and Families, the overarching goal of the grant is to implement a longitudinal curriculum inclusive of the content of the DATA 2000 waiver training that would enable all graduates to prescribe lifesaving medications for opioid use disorder.

With the closure of treatment clinics and a focus of emergency departments on COVID-19 patients during the pandemic [7], the virtual clinic was created with a dual mission of addressing service gaps while reducing face-to-face clinical interactions and providing students with experience in caring for PWID during these unprecedented times. Here, we describe the implementation of this student-run free virtual MOUD program (*TeleMOUD*) amid the COVID-19 pandemic in Miami, Florida.

## Methods

### Human subjects

This study was reviewed by the Institutional Review Board of the University of Miami (IRB # 20200879).

### Work flow

An online form was created for patients to request virtual appointments for MOUD, and these services were promoted via flyers at the IDEA SSP and on its social media accounts. Patients who submitted appointment requests are contacted by a student volunteer to collect paperwork confirming eligibility for the free clinic, which include Florida residency and income < 200% of the federal poverty level. An additional student volunteer assisted with the patient history and case presentation.

The existing clinic electronic health record (EHR) was updated with the Diagnostic and Statistical Manual of Mental Disorders, Fifth Edition (DSM-5) criteria for the diagnosis of opioid use disorder (OUD), and a modified Clinical Opiate Withdrawal Scale (COWS) [8] focused on the subjective symptoms of withdrawal. Appointments began with two students interviewing the patient through a remote videoconference platform or by phone. The students then discussed the case with the attending and re-connected with the patient as a group to complete the visit. The attending was responsible for checking the prescription drug monitoring program to evaluate for concurrent controlled substance prescriptions. Initially, paper MOUD prescriptions were provided, but this process was updated to electronic prescribing or phoning MOUD prescriptions to a mutually agreed upon pharmacy.

Students helped counsel patients on the logistics of buprenorphine induction and assisted patients as they went through the process. All patients started on MOUD had formal follow-up appointments scheduled within 2 weeks of initiation, every 2 weeks after that for 6 weeks, and thereafter every 4 weeks. Students remained in frequent contact with patients early in their treatment. Medication efficacy, craving management, and side effects were discussed and dosing titrated to desired effect by the physician.

### Statistical analysis

Data were collected retrospectively from the online appointment request system and clinic EHR. Frequency distributions for categorical variables and medians and interquartile ranges (IQR) were presented to describe the overall study sample. To understand the time intervals of the *TeleMOUD* care cascade, we calculated the mean number of days between steps.

## Results

### TeleMOUD care cascade

**Fig. 1:**
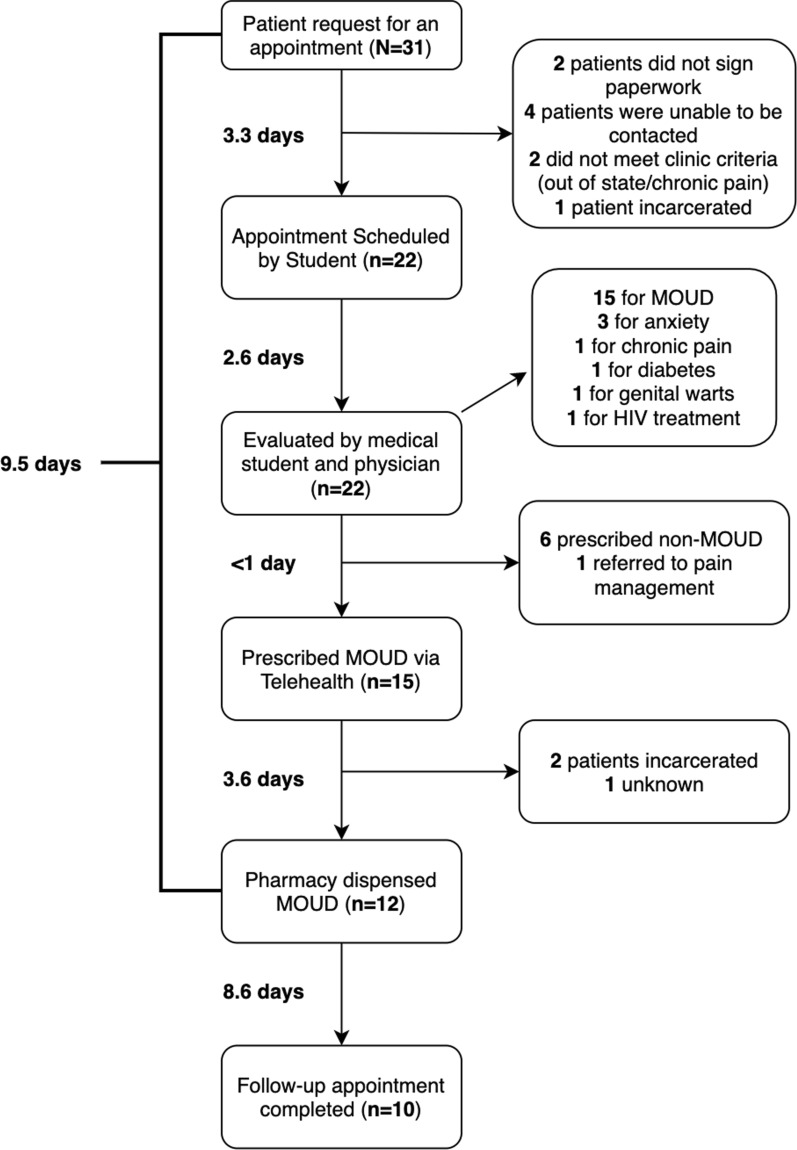
TeleMOUD care cascade. The steps between appointment request, completion of appointment, and follow-ups are displayed in descending boxes. Boxes on the right provide details on services provided or reasons for falling out of the care cascade. The mean number of days between each step is presented. *Of those who dropped out from MOUD dispensed to follow-up appointment, one person entered the hospital for behavioral and mental health services, and one person was lost to follow up

Between March 30, 2020, and June 08, 2020, 31 requests were made for telehealth services at the student clinic. Of these, 22 appointments were completed; reasons for the 9 non-completed appointments are described in Fig. 1. Of the patients with completed appointments, 15 (68%) were prescribed buprenorphine; 12 of 15 (80%) successfully picked up their prescription for buprenorphine at the pharmacy, and 10 of 12 (83%) completed their first follow-up visit. The average time from appointment request to receipt of medications was 9.5 days (Fig. 1).

### Characteristics of patients

The median age of patients was 35.5 years (Table 1), and the largest proportion of patients were white non-Hispanic (43%) and male (67%). Of the 15 patients seeking MOUD, 5 were currently injecting drugs, 2 used opioids by non-injection routes, 7 were refilling their prescription or changing providers, and 1 was already not currently using. Reasons for transition to *TeleMOUD* included temporary closure of treatment centers due to COVID-19, convenience of telehealth treatment, and dissatisfaction with their current provider.

**Table 1:** Descriptive statistics of those accessing telehealth during COVID-19 (*N* = 22)

Characteristic	*N* (%)
Age (median, IQR)	35.5 (33–51)
Biological sex	
Male	14 (67)
Female	7 (33)
Race/ethnicity	
White non-Hispanic	9 (43)
Black non-Hispanic	4 (19)
Hispanic	8 (38)
IDEA SSP participant	
Yes	11 (52)
No	10 (48)
Current injection drug use	
Yes	10 (50)
No	10 (50)
Substances injected (*n* = 10)	
Heroin	8 (80)
Fentanyl	5 (50)
Cocaine	2 (20)
Other (ketamine)	1 (10)
Self-reported HIV status	
Positive	3 (14)
Negative	16 (76)
Unknown	2 (10)
Self-reported HCV status	
Positive	8 (38)
Negative	10 (48)
Unknown	3 (14)

## Discussion

In this descriptive study, we demonstrate the feasibility of rapid implementation of a student-run free *TeleMOUD* program housed within an academic medical center-operated SSP. Despite multiple studies demonstrating the association of MOUD with reduced morbidity and mortality [9, 10], access to MOUD remains limited in the USA, with 96% of states reporting higher rates of opioid use than buprenorphine treatment capacity [11]. Due to the COVID-19 pandemic, this already limited access to MOUD was further impeded due to the closure of opioid treatment clinics and the prioritization of COVID-19 patients in hospital emergency departments [7].

Sexual Health Care Clinics and SSPs provided examples on how to connect patients with providers during COVID-19 via telehealth [12, 13], and a recent opinion paper demonstrated the utility of telehealth in preventing disruption of care for people with OUD and co-occurring mental health disorders during COVID-19 [14]. With the development of *TeleMOUD*, we aimed to mitigate disruptions in care by serving patients with OUD who could not receive in person care within a hospital or clinic setting. Additionally, we sought to provide medical students, who were mostly restricted from direct clinical care during the early stages of the pandemic, important experience treating patients with OUD while responding to a public health crisis.

There is a growing body of evidence demonstrating the utility of using telemedicine technology for treating OUD [15]. However, before the pandemic, adoption of telehealth for OUD had been limited by policies that required face-to-face interactions and monitoring of patients, technological challenges for patients and providers, and clinicians’ concerns about a negatively impacted doctor–patient relationship [16]. During the pandemic, *TeleMOUD* was possible because the Drug Enforcement Agency allowed for the initiation of buprenorphine treatment via telemedicine. Students proved key in helping to address technological issues by creating online appointment request forms, promoting the clinic via the SSP’s social media pages, and helping participants to sign forms virtually and access video conferencing apps. Providers and patients expressed great satisfaction with TeleMOUD, and the patients who saw a psychiatrist or mental health counselor, in conjunction with or following their MOUD provider, described this as a benefit.

Lack of medication availability at patient’s local pharmacies proved a challenge to both patients and providers, and this was a barrier to care. Cost of medication was a common concern for many patients, and students worked closely with them to obtain coupons that increased affordability. As a result of continuous process improvement efforts, we have now partnered with a pharmacy at a nearby safety-net healthcare system operating a 340B Drug Pricing Program that offers patients significant discounts irrespective of insurance status.

*TeleMOUD* became possible because Drug Enforcement Agency requirements for a face-to-face visit for the initiation of care and the prescription of controlled substances were lifted during the pandemic. Whereas we attempted to use videoconferencing technology for all visits, many of the patients served did not have access to appropriate technology and their visits were completed over the phone; reinstatement of the face-to-face requirement would pose a significant barrier to this model. Furthermore, the waiver of urine drug screens under the SAMHSA guidelines allowed us to rapidly implement this low barrier path to care initiation [17]; rules requiring urine drug screen for initiation of care should remain waived as an unnecessary burden to buprenorphine initiation. For insured patients, removal of onerous prior authorization requirements catalyzed rapid progress to a low-threshold MOUD model.

The implementation of a substance use disorder curriculum provided students with the knowledge to pursue and develop the TeleMOUD clinic, serve marginalized populations during the pandemic, and practice harm reduction. While it is too early to draw conclusions on the community impact of *TeleMOUD*, early evidence supports the implementation of a more robust *Tele-Harm Reduction* model at SSPs. Adapting educational protocols for telehealth across undergraduate medical education can illuminate to students the benefits and limitations of this technology, encourage innovation for the delivery of care, and prepare them to respond to future health crises. The policy changes that allowed these services to commence created new possibilities to reduce morbidity and mortality from preventable overdose among PWID, and it is our hope that beyond the COVID-19 era we will not forfeit the progress made during this pandemic.

## Data Availability

Not applicable.

## References

[1] Samuels EA (2020). Innovation during COVID-19: improving addiction treatment access. J Addict Med.

[2] Dunlop A (2020). Challenges in maintaining treatment services for people who use drugs during the COVID-19 pandemic. Harm Reduct J.

[3] Bartholomew TS, Nakamura N, Metsch LR, Tookes HE. Syringe services program (SSP) operational changes during the COVID-19 global outbreak. Int J Drug Policy. 2020;83. 10.1016/j.drugpo.2020.102821 PMC7290194 32591222

[4] Tookes H (2018). Rapid identification and investigation of an HIV risk network among people who inject drugs-Miami, FL. AIDS Behav.

[5] Ginoza ME (2020). Student-run free clinic at a syringe services program, Miami, Florida, 2017–2019.

[6] Glick SN, Prohaska SM, LaKosky PA, Juarez AM, Corcorran MA, Des Jarlais DC (2020). The impact of COVID-19 on syringe services programs in the United States. AIDS Behav..

[7] Khatri UG, Perrone J (2020). Opioid use disorder and COVID-19: crashing of the crises. J Addict Med.

[8] Wesson DR, Ling W (2003). The clinical opiate withdrawal scale (COWS). J Psychoact Drugs.

[9] Kimmel SD (2020). Association of treatment with medications for opioid use disorder with mortality after hospitalization for injection drug use-associated infective endocarditis. JAMA Netw Open.

[10] Sordo L, Barrio G, Bravo MJ, Indave BI, Degenhardt L, Wiessing L, Ferri M, Pastor-Barriuso R. Mortality risk during and after opioid substitution treatment: systematic review and meta-analysis of cohort studies. BMJ. 2017;357. 10.1136/bmj.j1550 PMC5421454 28446428

[11] Huhn AS, Dunn KE (2017). Why aren't physicians prescribing more buprenorphine?. J Subst Abuse Treat.

[12] Rogers BG (2020). Development of telemedicine infrastructure at an LGBTQ+ clinic to support HIV prevention and care in response to COVID-19, Providence, RI. AIDS Behav..

[13] Harris M (2020). Low barrier tele-buprenorphine in the time of COVID-19: a case report. J Addict Med.

[14] Lin LA, Fernandez AC, Bonar EE. Telehealth for substance-using populations in the age of coronavirus disease 2019: recommendations to enhance adoption. JAMA Psychiatry. 2020. 10.1001/jamapsychiatry.2020.1698 PMC8108064 32609317

[15] Lin LA (2019). Telemedicine-delivered treatment interventions for substance use disorders: a systematic review. J Subst Abuse Treat.

[16] Huskamp HA (2018). How is telemedicine being used in opioid and other substance use disorder treatment?. Health Aff.

[17] SAMHSA. FAQs: provision of methadone and buprenorphine for the treatment of opioid use disorder in the COVID-19 emergency. 2020. https://www.samhsa.gov/sites/default/files/faqs-for-oud-prescribing-and-dispensing.pdf. Cited 17 June 2020.

